# First Use of GORE TAG Thoracic Endograft with Active Control System in Traumatic Aortic Rupture

**DOI:** 10.1155/2020/3708287

**Published:** 2020-08-05

**Authors:** Fotios Eforakopoulos, Ioanna Akrida, Petros Zampakis, Konstantinos Katsanos, Panagiotis Papadopoulos, Efstratios Koletsis

**Affiliations:** ^1^Department of Cardiothoracic Surgery, University of Patras, Patras, Greece; ^2^Department of Surgery, University of Patras, Patras, Greece; ^3^Department of Radiology, University of Patras, Patras, Greece; ^4^Department of Interventional Radiology, University of Patras, Patras, Greece; ^5^Endovascular Clinical Specialist, Vista Medical SA, Athens, Greece

## Abstract

Patients after a high-velocity motor vehicle collision with rapid deceleration are at a significant risk of blunt aortic injury, a life-threatening condition that usually occurs in the aortic isthmus. Aortic transection is the second leading cause of death behind head injury for individuals aged 4 to 34. During the last two decades, there has been a shift from open towards the endovascular repair. Significant progress has been made recently in terms of the design of both the stent graft and the delivery system. We herein present the case of a female patient under dual antiplatelet therapy for coronary artery disease, with type IV blunt aortic injury (rupture) that was successfully repaired with Conformable Thoracic Endograft with Active Control System. This new device provides an intermediate deployment step at 50% and optional angulation control of the proximal part of the stent graft. These improvements are beneficial providing accurate device placement and maximum seal length in anatomies where the distal, as well as a proximal landing zone, is critical.

## 1. Introduction

Blunt aortic injury can be graded based upon CT findings as an intimal tear (type I), intramural hematoma (type II), pseudoaneurysm (type III), and rupture (type IV). Thoracic aortic injuries occur in approximately one-third of blunt traumatic deaths according to autopsy studies [1]. Most of the patients and more specifically 80% to 85% of them die before they can reach hospital care [1]. For the rest of the patients that do survive to reach a capable health care facility, there is a new trend in the treatment of choice that has emerged during the last two decades.

Open repair of traumatic rupture of the descending thoracic aorta is associated with a mortality rate of 24% to 42% [2]. The outcomes of untreated aortic rupture are overall poor. While awaiting repair, we aggressively control blood pressure and heart rate. Thoracic endovascular repair (TEVAR) has emerged as a reasonable alternative to open repair. TEVAR has been related to significantly lower mortality and spinal cord complication rates compared to open repair for traumatic thoracic aortic rupture [2]. However, the device-related complication rates have been documented to be as high as 20% in the important publication in 2008 by Demetriades et al. with the results of the AAST multicenter study [3]. There has been considerable improvement in medical care since this study. There is a significant evolution of TEVAR devices, more conformable and most suitable for blunt traumatic aortic ruptures, in comparison with devices that were first introduced for thoracic aortic aneurysms [4]. We herein present the case of a patient with a traumatic rupture of the descending thoracic aorta after a motor vehicle crash that was successfully repaired with GORE TAG Thoracic Endograft with Active Control System.

## 2. Case Report

A 57-year-old female patient was transferred to the University General Hospital of Patras from a secondary hospital, with the diagnosis of traumatic rupture of the descending thoracic aorta, after a high-velocity motor vehicle collision with two dead passengers on the site of the accident.

On the primary survey, she had airway patency, her breathing rate was 29 per minute, her oxygen saturation was 96% with the use of oxygen supplementation, she had 89 beats per minute, and her blood pressure was 115/70. She had a good level of consciousness, and her Glasgow Coma Scale was 15/15. Clinical examination showed bilateral tenderness over her anterior chest wall, with subcutaneous emphysema on the left, tenderness over her cervical and thoracic spine, as well as over both her scapulas and right humerus, and forearm fractures. She had no abdominal tenderness. She did not have any focal neurologic deficits. She had a chest tube on the left with small drainage of bloody fluid. From her routine laboratory tests on admission, her hemoglobin and hematocrit values were 8.1 g/dl and 23.4%.

The patient's medical history included an angioplasty for myocardial infarction one month ago, and she was under dual antiplatelet and b-blocker therapy.

She was immediately transferred into the operating theatre where she was subjected to endovascular repair of the traumatic aortic rupture. The operative findings were traumatic rupture of the descending aorta distal to the origin of the left subclavian artery, at the location of the aortic isthmus, with mediastinal hematoma and bilateral pleural effusion. A GORE TAG Thoracic Endograft with Active Control System (31 mm proximal, 26 mm distal) was placed through the right common femoral artery. The proximal edge of the endograft was placed distal to the left subclavian artery (zone 3). Intraoperative angiography demonstrated a good endograft position without endoleak and with the normal angiographic appearance of the left subclavian artery. Postoperative computed tomography confirmed these findings and also showed bilateral lung contusions with pleural effusion and multiple rib fractures, left pneumothorax with subcutaneous emphysema, bilateral scapula fractures, T4 spinal body fracture, multiple cervicals, and thoracic spinal transverse process fractures and minor injury of the superior splenic pole with small amount of free intra-abdominal fluid and suspicion of pseudoaneurysm in peripheral locations of the superior splenic pole. She was transferred to the Intensive Care Unit where she remained hemodynamically stable. Repeated computed tomography scan after 2 days showed good endograft position without endoleak, while no pseudoaneurysm suspicion of the superior splenic pole was raised in this exam (Figure S1). After she remained intubated for 10 days in the Intensive Care Unit, she was transferred to the cardiothoracic department from where she was discharged in good condition 12 days later, after repeated consultations from general surgeons, neurosurgeons, orthopedic surgeons, infectious diseases specialists, and psychiatrists. After one year of follow-up with CTA at 3, 6, and 12 months, the patient remains in excellent clinical condition.

## 3. Discussion

Since its introduction by Dake et al. in the 1990s [5], thoracic endovascular aortic repair (TEVAR) has become the modality of choice for the treatment of thoracic aortic pathologies. The TAG device is a self-expanding device supported by a nitinol exoskeleton lined with expanded polytetrafluoroethylene (ePTFE) and covered with ePTFE and fluorinated ethylene propylene (FEP) in order to decrease the possibility for type IV endoleak. Conformable TAG graft (CTAG) has a sheathless delivery system for conforming to smaller, more tortuous, and/or tapered thoracic aortic anatomy. Despite recent advances, complications such as windsocking and bird-beaking of the stent could not be avoided, especially in the aortic arch. The inability to conform to arch anatomy leads to type I endoleak, a life-threatening situation. Increased blood pressure and flow velocity in the arch demand enhanced deployment accuracy in order to avoid the windsocking complication. In order to debilitate these challenges, the novel Conformable GORE TAG Device has a new nested handle delivery system that allows staged, predictable endograft deployment and in situ angulation control of the proximal end of the stent graft. Simultaneously, the conformable graft enables accurate stent apposition with sutureless attachment, oversizing windows that facilitate optimized radial fit and respond to anatomic requirements. Staged deployment allows the device to be first opened to an intermediate diameter while ensuring continuous blood flow. The next deployment step expands the device to its full diameter. This feature allows for hemodynamic stability throughout the entire procedure. The second deployment sequence to full diameter from trailing to leading ends, followed by the attachment of the stent graft to the catheter via the lock wire, guarantees full control during the entire deployment process. The angulation feature can be used at both intermediate and full diameter stages. By nesting the stent rows along the inner curve of the aorta, angulation achieves orthogonal stent placement in tight arches. The angulation control dial of the device remains accessible even after full expansion, and the user has a new opportunity for optional angulation at full diameter [6].

To the best of our knowledge, it is the first time that the use of CTAG Device with Active Control System has been reported in the literature in blunt aortic injury and particularly in aortic rupture. A literature search was done via the internet in search engines of PubMed and Google Scholar with keywords: aortic rupture, conformable CTAG Active Control System. There was no report of the use of this stent in blunt aortic injury and especially in aortic rupture. This endograft showed excellent deployment accuracy and conformability. Its new characteristics such as staged deployment and angulation control provide precise positioning preventing bird-beak effect, and it was preferably chosen for cases with challenging and short distal landing zones [7]. Ease of use and conformability of the device may be of great assistance in blunt aortic injuries, conditions that require optimal placement and accuracy especially in younger patients with multiple severe injuries.

## 4. Conclusion

GORE TAG Conformable Thoracic Stent with Active Control System showed excellent deployment accuracy and conformability in a challenging case of traumatic aortic rupture. Its unique features of staged deployment and angulation control provide extremely convincing and promising results. However, large studies and longer follow-up are needed to confirm our observations. We expect that the SURPASS registry will respond to these endpoints.

## Supplementary Materials

Supplementary Materials Figure S1: postoperative CTA demonstrating successful exclusion of the aortic transection with optimal device placement at both proximal and distal landing zones.
