# Supplementary material for: First Use of GORE TAG Thoracic Endograft with Active Control System in Traumatic Aortic Rupture
Source: Case Rep Surg. 2020 Aug 5;2020:3708287. doi: 10.1155/2020/3708287 (PMC7426760; doi:10.1155/2020/3708287)
Supplement: Supplementary Materials — Figure S1: postoperative CTA demonstrating successful exclusion of the aortic transection with optimal device placement at both proximal and distal landing zones. [file 3708287.f1.doc]

Figure S1: Postoperative CTA demonstrating successful exclusion of the aortic transection with optimal device placement at both proximal and distal landing zones.
